# S100A4 Protects Myeloid-Derived Suppressor Cells from Intrinsic Apoptosis *via* TLR4–ERK1/2 Signaling

**DOI:** 10.3389/fimmu.2018.00388

**Published:** 2018-03-05

**Authors:** Qingcui Li, Chengliang Dai, Rui Xue, Peigang Wang, Lin Chen, Yijie Han, Ulrike Erben, Zhihai Qin

**Affiliations:** ^1^Medical Research Center, The First Affiliated Hospital of Zhengzhou University, Zhengzhou, China; ^2^Key Laboratory of Protein and Peptide Pharmaceuticals, CAS Center for Excellence in Biomacromolecules, Chinese Academy of Sciences-University of Tokyo Joint Laboratory of Structural Virology and Immunology, Institute of Biophysics, Chinese Academy of Sciences, Beijing, China

**Keywords:** S100A4, myeloid-derived suppressor cells, intrinsic apoptosis, toll-like receptor-4, ERK1/2 signaling

## Abstract

Myeloid-derived suppressor cells (MDSCs) often expand during cancer or chronic inflammation and dampen immune responses. However, mechanisms underlying their capacity to escape intrinsic apoptosis in the inflammatory environment are still largely unknown. In this study, we investigated this in mouse tumor models with MDSC accumulation. Spontaneous rejection of tumors implanted into mice deficient for the small Ca^2+^-binding protein S100A4 (S100A4^−/−^) was accompanied by low numbers of peripheral MDSCs. This was independent of S100A4 expression on tumor cells. In contrast, MDSCs from S100A4^−/−^ tumor-bearing mice showed a diminished resistance to the induction of intrinsic apoptosis. Further studies demonstrated that S100A4 protects MDSCs from apoptosis through *toll*-like receptor-4/extracellular signal-regulated kinase-dependent caspase-9 inhibition. The finding that S100A4 is critical for MDSC survival in inflammatory environments might have important implications for the clinical treatment of cancer or inflammation-related diseases.

## Introduction

Myeloid-derived suppressor cells (MDSCs) accumulate in chronic inflammatory states such as the conditions associated with cancer (1–4). Multiple pro-inflammatory factors such as GM-CSF (5), VEGF (6), TNF-α (7, 8), interleukin-1β (9), and interferon-γ (10) have been identified as mediators of MDSC accumulation. In contrast to the understanding of MDSC induction, little is known about the conditions or factors that control their apoptosis. These cells must effectively protect themselves from apoptosis in the pro-inflammatory environment. Here, we focus on mechanisms involved in protecting MDSCs from strongly proapoptotic environments.

Apoptosis regulates the persistence of MDSCs during inflammation. Moreover, MDSCs are more sensitive to apoptosis due to increased levels of death receptors such as Fas (11). MDSCs exert cytotoxicity by producing reactive oxygen species (ROS), nitric oxide (NO), and endoplasmic reticulum (ER) stress (12). Actually, the inducible NO synthase activity in MDSCs is instrumental to suppress T-cell function (13). The fact that MDSCs accumulate in large numbers suggests that these cells have mechanisms to protect them from apoptosis (7, 11, 14, 15). We previously showed that activation of the TNF receptor-2 pathway counteracts the proapoptotic effect of TNF on MDSCs by enhancing cellular inhibition of the activity of caspase-8, the checkpoint enzyme required for the induction of extrinsic apoptosis upstream of caspase-3 (7). Intriguingly, high levels of ROS or NO, as well as ER stress induced by MDSCs, preferentially activate intrinsic apoptosis through caspase-9, rather than extrinsic apoptosis (16–20), but how MDSCs survive these non-specific threats is not well understood.

S100A4 is a member of the S100 family (21) and has both intracellular and extracellular functions. Inside the cell, it interacts with cytoskeletal proteins involved in cell motility (22). S100A4 can also be secreted from cells *via* an unknown mechanism to mediate signaling through multiple cell surface receptors including receptor of advanced glycation end-products (RAGE) (23) and toll-like receptor-4 (TLR4) (24). Intra- and extracellular S100A4 participates in cell survival and migration or angiogenesis (25). We were intrigued by the previous observation of high serum and tissue levels of S100A4 in cancer and chronic inflammation (22, 26, 27), and we thus hypothesized that exogenous S100A4 might support MDSC accumulation under inflammatory conditions.

We found that low peripheral MDSCs accompanied the rejection of S100A4-positive or S100A4-negative tumors implanted into S100A4-deficient mice (S100A4^−/−^). Caspase-9, but not caspase-8, was activated in the MDSCs from S100A4^−/−^ mice. Proving its crucial role in the induction of intrinsic apoptosis, exogenous S100A4 directly abrogated the effect of 5-fluorouracil (5-FU) *in vitro*. The use of a TLR4-deficient mouse tumor model, as well as *in vitro* experiments in which the S100A4 receptor was blocked in MDSCs, finally established that the activation of TLR4–ERK signaling by extracellular S100A4 is responsible for the resistance of MDSCs to intrinsic apoptosis induction. These results, suggesting a new function for an “old” molecule, define S100A4 as an important survival factor for MDSCs and imply that it could represent a novel therapeutic target for inflammation-related diseases.

## Materials and Methods

### Mice

S100A4^−/−^ and TLR4^−/−^ mice in a C57BL/6 background were purchased from Jackson Laboratory (Bar Harbor), and C57BL/6 wild-type (WT) mice were purchased from Vital River (Beijing). All mice were bred under specific pathogen-free conditions and female mice aged 6–8 weeks were used for the experiments. All animal experiments were approved by the Animal Care and Use Committee of the Institute of Biophysics, Chinese Academy of Sciences Beijing (Protocol no. SYXK2014-34).

### Cell Lines

The immortalized MSC2 MDSC cell line was generously provided by the François Ghiringhelli lab (28). MCA205, a cell line from 3-methylcholanthrene-induced fibrosarcoma in C57BL/6 mice, was generated as described previously (29). The B16F10 melanoma, Lewis lung cancer (LLC), and Sp2/0 myeloma cell lines were purchased from the American Type Culture Collection (LGC Standards). Cells were routinely cultured in DMEM (or RPMI1640 for Sp2/0 and MSC2) supplemented with 10% FCS, 100-U/mL penicillin, and 100-U/mL streptomycin (all from Gibco). Two days before experiments, MSC2 cells were precultured with 100 ng/mL interleukin-4 (R&D) to ensure immunosuppressive capacity as previously described (30).

### *In Vivo* Tumor Induction

Exponentially growing tumor cells, grown in culture, were harvested and washed; 5 × 10^5^ cells in 200 µL phosphate-buffered saline (PBS) were subcutaneously injected into the abdominal region of mice. Starting at day 7 after tumor-cell inoculation, tumor growth was monitored every 2–3 days, and tumor volumes (*V*) were assessed in mm^3^ using the formula: *V* = 0.5 (*a* × *b*^2^) with *a* being the long and *b* the short diameters of the tumor.

### Preparation of Primary MDSCs

Splenic MDSCs were prepared as described previously (7). Briefly, mice were subcutaneously injected with 5 × 10^5^ MCA205 tumor cells. When tumors reached a volume of at least 1,000 mm^3^ (at around day 17), single-cell suspensions from the spleens of tumor-bearing mice were fractionated by Percoll density-gradient centrifugation (Beckman). CD11b^+^GR1^+^ cells were subsequently isolated using a MDSC Isolation Kit for positive selection according to the manufacturer’s protocol (Miltenyi Biotec). Cell recovery from spleens of tumor-bearing S100A4^−/−^ mice was typically about 5–10% lower than that of WT counterparts.

### Flow-Cytometric Analysis

Single-cell suspensions prepared from bone marrow, peripheral blood, spleen, or tumor tissue were stained with directly labeled mouse-specific monoclonal antibodies that were purchased from Biolegend, including those specific for CD4 (RM4-5), CD8 (53–6.7), CD11b (M1/70), GR1 (RB6-8C5), Ly6G (1A8), Ly6C (HK1.4), B220 (RA3-6B2), NK1.1 (PK136), FOXP3 (MF-23), CD11c (N418), and cleaved-caspase-3 (5A1E). Before staining for FOXP3 and cleaved caspase-3, cells were fixed and permeabilized according to the manufacturer’s instructions (eBioscience). Apoptotic cells were marked by the Annexin V Apoptosis Detection Kit (Biolegend) or by terminal deoxynucleotidyl transferase dUTP nick end labeling (TUNEL) (Beyotime) according to the manufacturers’ protocols. Using a FACS Calibur device (BD Biosciences), 100,000 events were recorded for each sample, and data were analyzed with FACS Diva (BD Biosciences) and FlowJo software (Tree Star). Numbers in dot or histogram plots refer to the percentages of positive cells.

### *In Situ* Immunofluorescence Staining

Tumor and spleen tissues from MCA205 tumor-bearing mice were fixed and prepared for cryostat sections as previously described (31). Cultured MSC2 cells were fixed with 4% paraformaldehyde for 10 min on ice, washed three times with PBS, and treated with hydrogen peroxide for 30 min at 4°C. Tissue sections (7 µm for tumors; 5 µm for spleens) were incubated with rat anti-mouse CD11b (M1/70) or GR1 (RB6-8C5; both BD Biosciences); MSC2 cells seeded on sterile glass coverslips were used for staining. After fixation, permeabilization, and blocking, cells were incubated with rat anti-mouse cleaved caspase-3 (5A1E, CST). Primary antibody binding was detected using Alexa Fluor 555 goat anti-rat (#1722994) or Alexa Fluor 488 goat anti-rabbit (#1672238) antibodies (both from Life Technologies). Nuclei were counterstained with 4’,6-diamidino-2-phenylindole (DAPI; Sigma-Aldrich). Primary antibodies were omitted for negative control samples. Slides were mounted for fluorescence observation using an FV1000 confocal microscope and FV10-ASW1.7 Viewer software (both Olympus).

### T-Cell Proliferation Assays

Freshly prepared splenocytes from tumor-free WT mice were labeled with 2.5-mM carboxyfluorescein (CFSE; Sigma) for 10 min at 37°C before 3 × 10^5^ cells in 100-µL culture medium containing 2.5-mg/mL concanavalin A (Sigma) were distributed in 96-well round-bottom plates. Cells were cultured with or without 1 × 10^6^ CD11b^+^Gr1^+^ MDSCs freshly prepared from MCA205 tumor-bearing mice. After 72 h, cells were collected and stained, and the dilution of CFSE in CD4^+^ or CD8^+^ T cells was determined by flow-cytometric analysis.

### *In Vitro* Induction and Blocking of Apoptosis

In brief, 5 × 10^5^ MCS2 cells were incubated with 80-nM 5-FU (Sigma) for 24 h. For blocking experiments, 5 × 10^5^ MCS2 cells were pretreated with the appropriate reagent for 30 min at 37°C. TLR4 was specifically inhibited by 10-µM VIPER (Novus) (32), RAGE by 100-nM FPS-ZM1 (Millipore) (33), and ERK1/2 by 50-nM SCH772984 (ChemCatch).

### Transmission Electron Microscopy

Treated MSC2 cells (5 × 10^5^) were prefixed in 2.5% glutaraldehyde, postfixed in 1% osmic acid, dehydrated in gradient acetone, and embedded in epoxy resin (Agar Scientific). Ultrathin sections (60 nm) were cut, stained with lead citrate, and assessed for morphological changes using a transmission electron microscope (Olympus).

### Western-Blot Analysis

Cells were lysed with RIPA solution [50-mM Tris–HCl (pH 7.5), 150-mM NaCl, 1.0% Nonidet P-40, 0.5% sodium deoxycholate, 0.1% SDS, 1-mM EDTA] supplemented with 100-µM phenylmethane-sulfonyl fluoride, 25-µg/mL aprotinin, 1-mM sodium orthovanadate, and 50-mM NaF. Proteins from cell extracts were separated using homogenous 10% SDS-PAGE and transferred to nitrocellulose membranes (GE Healthcare).

The following monoclonal and polyclonal primary anti-mouse antibodies were purchased from Cell Signaling Technology: cleaved caspase-3 (5A1E), cleaved caspase-8 (D5B2), cleaved caspase-9 (#9509), BAX (#2772), cytochrome c (D18C7), β-actin (13E5), p-NF-κB p65 (pp65; 7F1), NF-κB (p65; C22B4), p-ERK1/2 (D13.14.4E), ERK1/2 (3A7), p-AKT (D9E), AKT (40D4), p-p38 (D3F9), and p38 (L53F8). Peroxidase-conjugated goat anti-mouse (#32430) or goat anti-rabbit (#32460) antibodies (both from Thermo Fisher) were used as secondary antibodies. Specific bands were visualized using a chemiluminescent substrate (Thermo), which was detected using a Chemiluminescence Imaging System (Clinx).

### Real-time RT-PCR

Total RNA was extracted from 1 × 10^7^ isolated cells and quantified using a ND-1000 spectrophotometer (NanoDrop Technologies). The cDNA, synthesized from 2-µg RNA using 9-nucleotide random primers (TaKaRa) and M-MLV reverse transcriptase (Promega), was subjected to real-time PCR using the SYBR Green Mixture Kit (Genstar) according to the manufacturer’s instructions and specific primers with a Rotor-GeneTM6000 (Corbett). The mouse-specific primers were as follows: *Bid3* forward: 5′-GAAACCCTGTGTCCTTGGAG-3′, reverse: 5′-TGTCTGTGTTTCCACCATCA-3′; *Bad* forward: 5′-GAGGAGGAGCTTAGCCCTTT-3′, reverse: 5′-AGGAACCCTCAAACTCATCG-3′; *Bak1* forward: 5′-ATATTAACCGGCGCTACGAC-3′, reverse: 5′-AGGCGATCTTGGTGAAGAGT-3′; *Bax* forward: 5′-TAGCAAACTGGTGCTCAAGG-3′, reverse: 5′-TCTTGGATCCAGACAAGCAG-3′; *Casp9* forward: 5′- CACAGCAAAGGAGCAGAGAG-3′, reverse: 5′- TCTGAGAACCTCTGGCTTGA-3′; *Actb* forward: 5′-ACATCTGCTGGAAGGTGGAC-3′, reverse: 5′-GGTACCACCATGTACCCAGG-3′. Individual expression levels were calculated relative to those of *Actb*.

### Caspase-3 and Caspase-9 Activity

Caspase activity was assessed using a colorimetric assay kit according to the manufacturer’s instructions (Beyotime). Briefly, 1 × 10^6^ CD11b^+^GR1^+^ cells were freshly isolated from MCA205 tumor-bearing mice, or MSC2 cells were used after 5-FU and S100A4 treatment. Cell lysates (10 µL) and 80 µL of reaction buffer [1% NP-40, 20-mM Tris–HCl (pH 7.5), 137-mM nicotinamide adenine dinucleotide, and 10% glycerol] containing 10-µL caspase-3 substrate (Ac-DEVD-pNA, 2 mM) or caspase-9 substrate (Ac-LEHD-pNA, 2 mM) were incubated in 96-well flat-bottom plates at 37°C for 4 h. The optical density was measured at 405 nm using a microplate reader (Bio-Rad Laboratories).

### Statistics

The data were analyzed using GraphPad Prism software (version 5; GraphPad). *P* < 0.05, as determined by a Mann–Whitney *U* test (Mann–Whitney) or a one-way ANOVA performed on ranks with subsequent Dunn’s posttests (ANOVA), was considered statistically significant.

## Results

### Attenuated Tumor Growth in S100A4^−/−^ Mice Associated with Small Proportions of MDSCs

**Figure 1:**
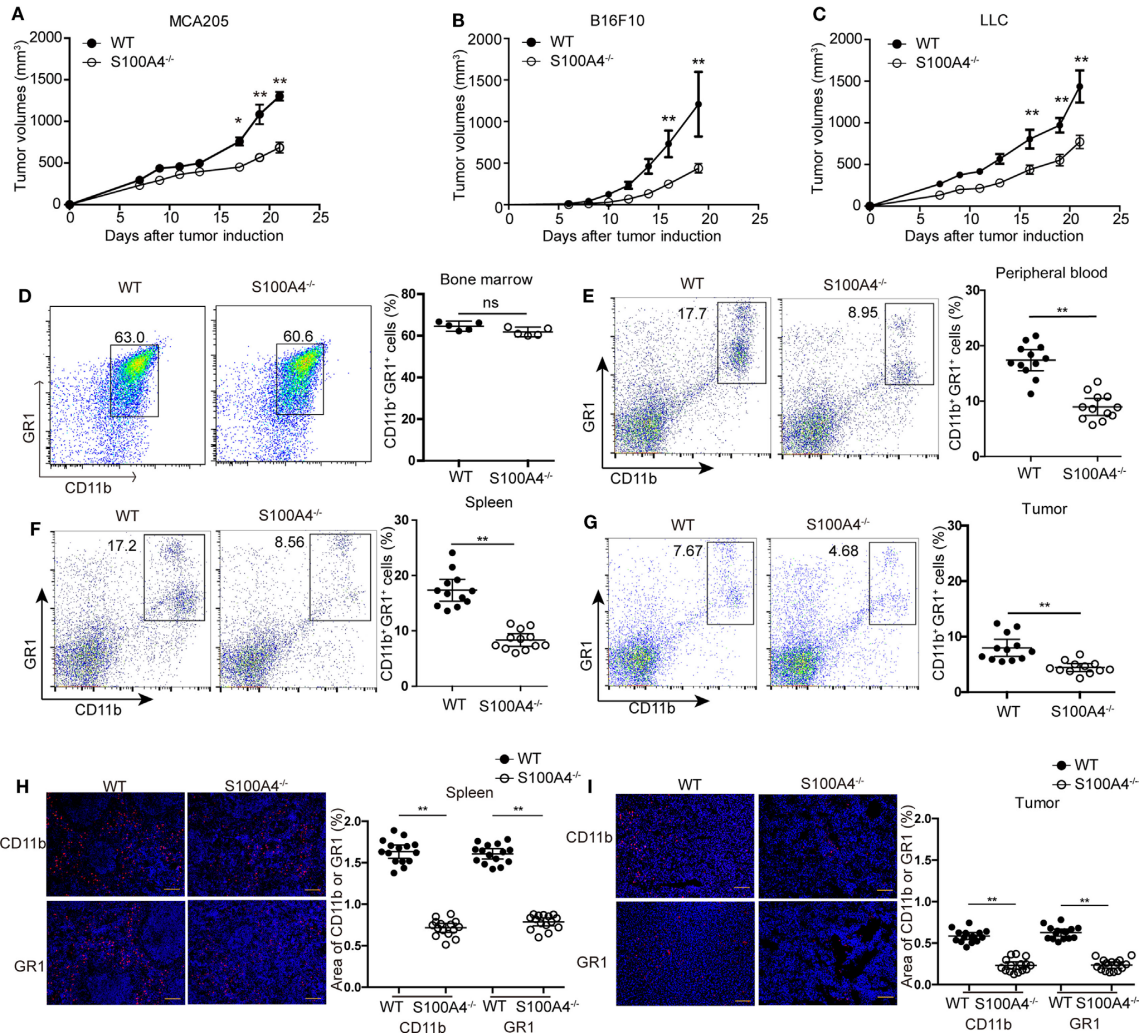
Tumor growth and accumulation of myeloid-derived suppressor cells (MDSCs) in S100A4^−/−^ mice. Wild type (WT) and S100A4^−/−^ mice were subcutaneously injected with 5 × 10^5^ **(A)** MCA205, **(B)** B16F10, or **(C)** Lewis lung cancer (LLC) tumor cells. Tumor volumes were monitored over time after tumor-cell inoculation. Results represent three independent experiments; mean ± SEM, *n* = 5 mice per group. *P < 0.05, **P < 0.01, and Mann–Whitney. **(D–I)** WT and S100A4^−/−^ mice were subcutaneously injected with 5 × 10^5^ MCA205 cells. Seventeen days after tumor-cell inoculation cells isolated from **(D)** bone marrow, **(E)** peripheral blood, **(F)** spleen, or **(G)** tumors were stained for CD11b and GR1 and assessed by flow cytometry. Percentages of CD11b^+^GR1^+^ MDSCs relative to total cells are given. Shown is combined raw data from three independent experiments. Mean and 95% CI, *n* = 5–12 mice per group; **P < 0.01, Mann–Whitney. **(H,I)** CD11b^+^ and GR1^+^ cells in **(H)** spleen or **(I)** tumor sections of WT and S100A4^−/−^ mice 17 days after tumor-cell inoculation were visualized by immunofluorescence staining (red). Nuclei were counterstained with DAPI. Original magnification, × 100. Scale bars: 100 µm. The area of CD11b or GR1 staining was analyzed by ImageJ software. Combined raw data from three independent experiments are shown. Mean and 95% CI, 15 sections from *n* = 5 mice per group. **P < 0.01, Mann–Whitney.

First, we investigated the effect of host S100A4 on the growth of implanted tumors. WT and S100A4-deficient (S100A4^−/−^) mice were inoculated with cells of the MCA205 fibrosarcoma cell line. Seventeen days after tumor-cell inoculation, tumors in S100A4^−/−^ mice were significantly smaller than tumors in WT control mice. At day 21, tumor volumes in WT mice were approximately twofold greater than those in S100A4^−/−^ mice (Figure 1A). To test whether the inhibitory effect of host S100A4 deficiency on tumor growth was specific for the MCA205 fibrosarcoma cell line, tumors derived from B16F10 melanoma or LLC Lewis lung carcinoma cell lines were utilized in the same model. Similar to results with MC205 cells, reduced tumor growth was observed in S100A4^−/−^ mice compared with that in WT mice with B16F10- or LLC-derived tumors; this became significant 17 days after tumor-cell inoculation and the differences increased until the end of the experiments (Figures 1B,C). MCA205 and LLC cells, but not B16F10 tumor cells, expressed the S100A4 protein (Figure S1A in Supplementary Material).

Changes in tumor burden independent of the presence of S100A4 in the implanted tumor cells implied that host S100A4 is predominantly involved in inhibiting tumor growth in S100A4^−/−^ mice. We further studied this using the MCA205 tumor model.

### Tumor-Bearing S100A4^−/−^ Mice Impairing Capacity to Accumulate Peripheral MDSCs

We studied immune-cell types at the time point at which differences in tumor sizes between WT and S100A4^−/−^ mice first became apparent. Percentages of CD11c^+^ dendritic cells, B220^+^ B cells, CD4^+^ and CD8^+^ T cells, forkhead box protein (FoxP3^+^) regulatory T cells, and NK1.1^+^ natural killer (NK) cells were the same in spleens of MCA205 tumor-bearing WT and S100A4^−/−^ mice (Figure S2 in Supplementary Material). The proportions of CD11b^+^GR1^+^ MDSCs cells in the bone marrow of both mouse strains were also comparable (Figure 1D). However, proportion of CD11b^+^GR1^+^ MDSCs in the peripheral blood (Figure 1E), spleen (Figure 1F), and in tumors (Figure 1G) of S100A4^−/−^ mice were only half that of their WT counterparts.

Immunofluorescence staining *in situ* for CD11b^+^ and GR1^+^ confirmed the quantitative findings of the aforementioned flow-cytometric analysis. These cells were evenly scattered in the red pulp outside the germinal centers within the spleen (Figure 1H) or throughout the tumor tissues (Figure 1I). MDSCs did not form locally restricted aggregates, and the distribution patterns did not depend on the presence of S100A4.

**Figure 2:**
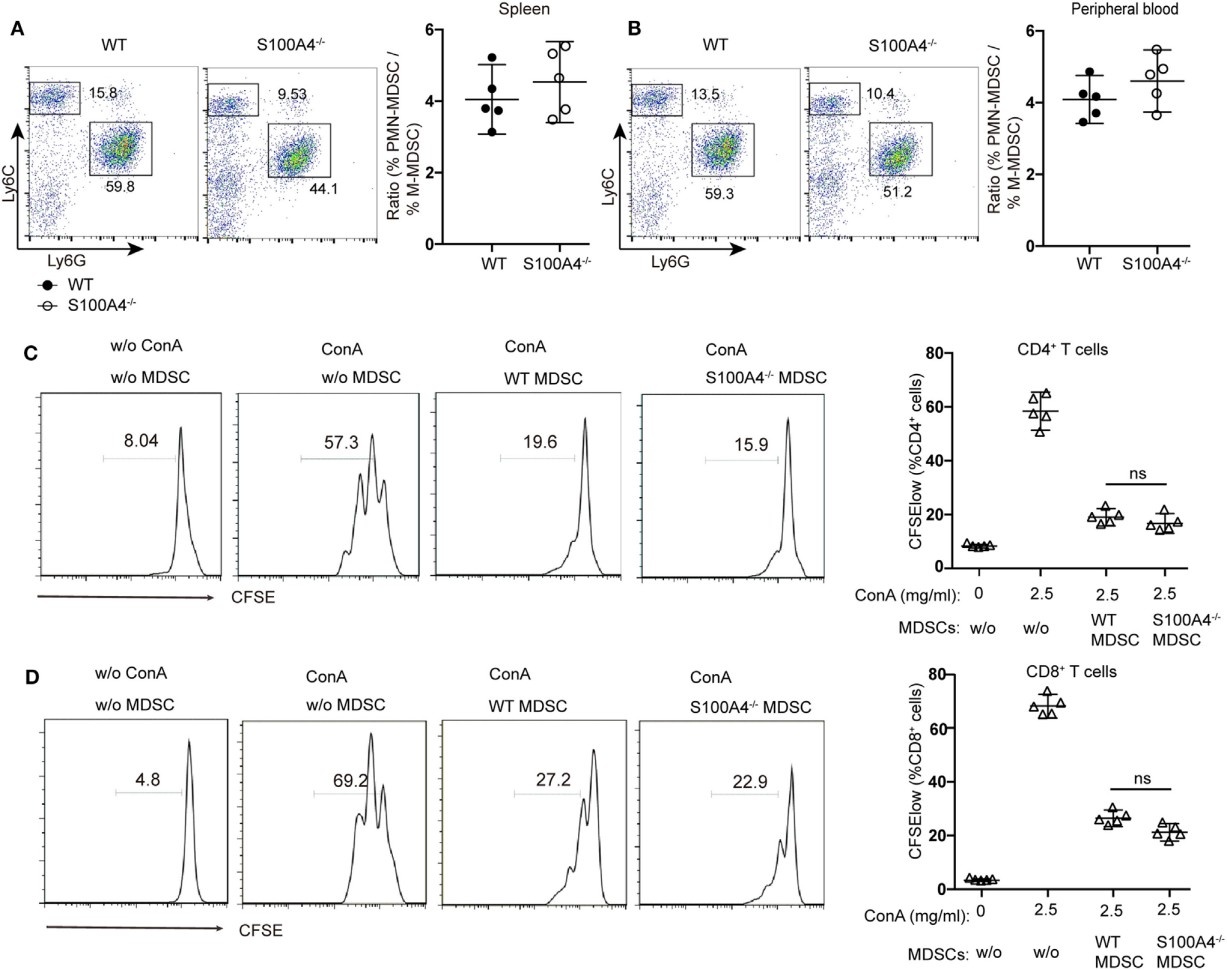
Functionality of myeloid-derived suppressor cells (MDSCs) in S100A4^−/−^ mice. **(A,B)** wild type (WT) and S100A4^−/−^ mice were subcutaneously injected with 5 × 10^5^ MCA205 cells. Seventeen days after tumor-cell inoculation, cells isolated from **(A)** spleen or **(B)** peripheral blood, stained for CD11b, Ly6C, and Ly6G, were assessed by flow cytometry. Ratios of CD11b^+^Ly6C^hi^Ly6G^−^ monocytic (M−)MDSCs and CD11b^+^Ly6C^low^Ly6G^+^ polymorphonuclear (PMN-)MDSCs are given. Representative results of three independent experiments are shown. Mean and 95% CI, *n* = 5 mice per group; **P < 0.01, Mann–Whitney. **(C,D)** Carboxyfluorescein (CFSE)-labeled tumor-free WT splenocytes were stimulated with concanavalin A. These cells were cultured with or without CD11b^+^GR1^+^ cells (1.5 × 10^4^) derived from the spleens of tumor-bearing WT or S100A4^−/−^ mice. After 72 h, collected cells were stained for CD4 and CD8. T-cell proliferation was determined by CFSE dilution in gated CD4^+^or CD8^+^T cells. Combined raw data from three independent experiments are shown. Mean and 95% CI, *n* = 5 mice per group; **P < 0.01, Mann–Whitney.

Investigating the effect of defined MDSC subpopulations, we found that the decreases in CD11b^+^Ly6C^hi^Ly6G^−^ monocytic MDSCs or CD11b^+^Ly6C^low^Ly6G^+^ polymorphonuclear MDSCs from S100A4^−/−^ mice were comparable in the spleen (Figure 2A) and peripheral blood (Figure 2B). Therefore, GR1, comprising Ly6C and Ly6G, could sufficiently differentiate the MDSC population and we did not further distinguish between these two subpopulations.

To test whether S100A4 deficiency affects the immunosuppressive function of MDSCs, CD11b^+^GR1^+^ cells isolated from the spleens of WT and S100A4^−/−^ tumor-bearing mice were cocultured with T cells. CFSE dilution within the T-cell population confirmed that MDSCs from WT or S100A4^−/−^ mice exhibited similar strong capacity to inhibit the proliferation of CD4^+^ (Figure 2C) and CD8^+^ T cells (Figure 2D).

Therefore, we did not attribute the reduced tumor growth in S100A4-deficient mice to impaired immunosuppressive capacity of the S100A4^−/−^ MDSCs but to impaired MDSC accumulation.

### Enhanced Apoptosis Responsible for Reduced MDSC Accumulation in S100A4^−/−^ Mice

**Figure 3:**
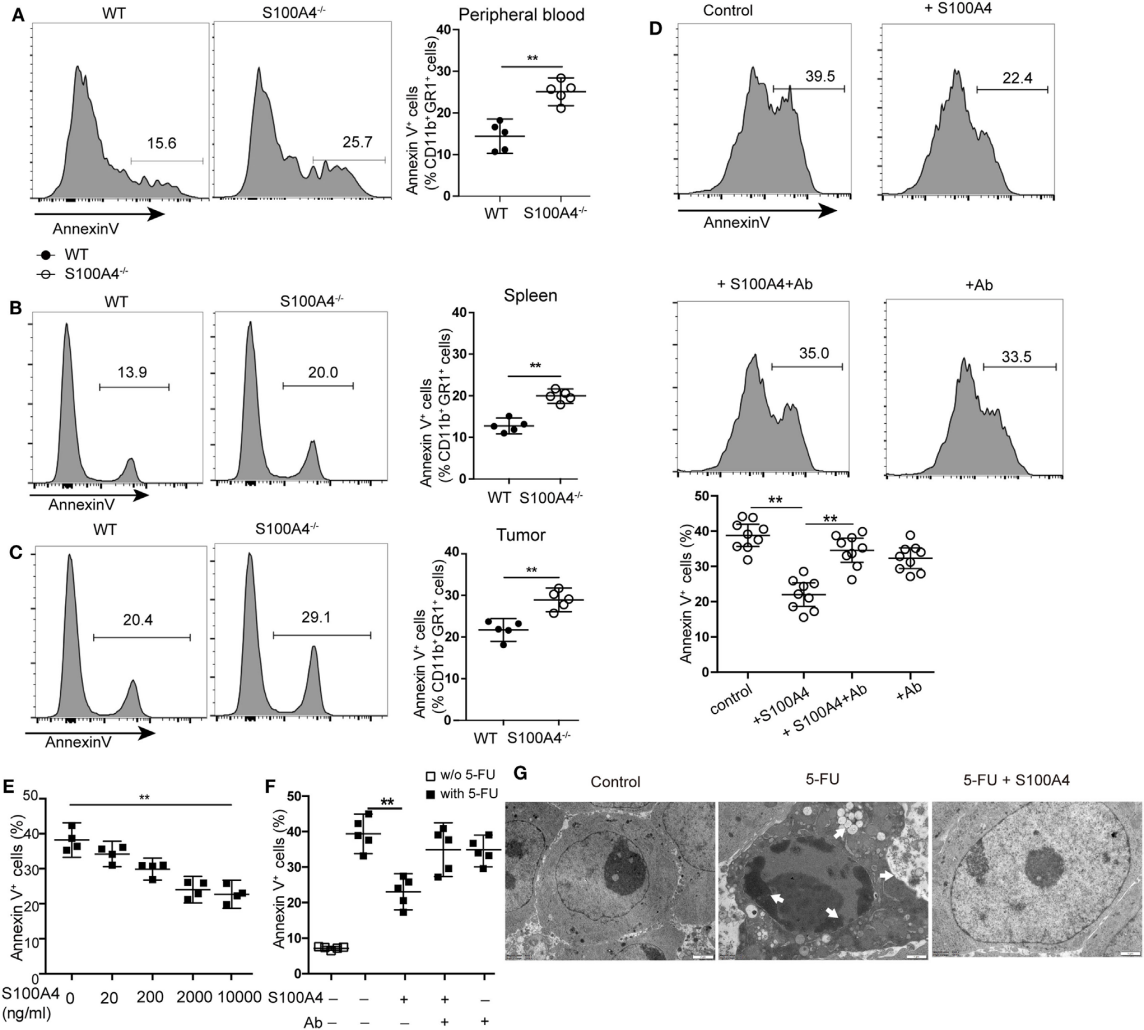
Extracellular S100A4 protecting myeloid-derived suppressor cells (MDSCs) from apoptosis. **(A)** Peripheral blood cells, **(B)** splenocytes, or **(C)** tumor cells were isolated from wild type (WT) and S100A4^−/−^ mice and stained for CD11b, GR1, and exposed phosphatidylserine. The proportion of Annexin V^+^ cells within the CD11b^+^GR1^+^ population was detected by flow cytometry. **(A–C)** Representative results of three independent experiments are shown. Mean and 95% CI, *n* = 5 mice per group; **P < 0.01, Mann–Whitney. **(D)** MDSCs isolated from S100A4^−/−^ mice were cultured for 24 h in the presence of recombinant S100A4 (1 µg/mL) alone or with S100A4 preincubated with the 3B11 antibody (6 µg/mL). The proportion of Annexin V^+^ cells within the CD11b^+^GR1^+^ population was detected by flow cytometry. Combined raw data from three independent experiments are shown. Mean and 95% CI, *n* = 9 per group; **P < 0.01, Mann–Whitney. **(E)** MSC2 cells were pretreated with 100 ng/mL of IL-4 for 2 days. MSC2 cell apoptosis induced by 5-fluorouracil (5-FU) alone or in the presence of increasing concentrations of S100A4 was assessed by Annexin V-binding. Representative results of three independent experiments are shown. Mean and 95% CI, *n* = 4 per group; **P < 0.01, Mann–Whitney. **(F)** MSC2 cells were treated for 24 h with 5-FU alone or in the presence of recombinant S100A4 (1 µg/mL) and the neutralizing antibody 3B11 (6 µg/mL). Annexin V-binding was evaluated by flow cytometry. Representative results of three independent experiments are shown. Mean and 95% CI, *n* = 5 per group; **P < 0.01, Mann–Whitney. **(G)** MSC2 cell morphology was studied by transmission electron microscopy. Cells were left untreated (control) or were incubated with 5-FU alone or in combination with S100A4 for 24 h. Arrows point to karyopyknosis, to undulations in the plasma membrane, and to vacuoles. Representative results of three independent experiments are shown; *n* = 4 per group. Scale bars: 1 µm.

Increased apoptosis was hypothesized to be the reason for overall reductions of peripheral MDSCs in S100A4^−/−^ mice. Apoptosis analysis revealed a significant increase of Annexin V^+^ CD11b^+^GR1^+^ cells in S100A4^−/−^ mice compared with that in WT mice in peripheral blood (Figure 3A), spleen (Figure 3B), and tumors (Figure 3C).

To investigate whether S100A4 directly protects MDSCs from apoptosis, freshly isolated MDSCs from spleen cells of S100A4^−/−^ mice were cultured in the presence of recombinant S100A4. After 24 h of *in vitro* culture, apoptosis in primary S100A4^−/−^ MDSCs was significantly reduced with recombinant S100A4. Preincubating S100A4 with the S100A4-neutralizing monoclonal antibody 3B11 (see Data Sheet 1 in the Supplementary Material) abolished the protection of primary MDSCs by exogenous S100A4, whereas the antibody alone did not alter MDSC apoptosis (Figure 3D).

To determine how S100A4 prevents apoptosis in MDSCs, we utilized the murine MDSC-derived cell line MSC2 for *in vitro* experiments. MSC2 cells were pretreated with 100 ng/mL of IL-4 for 2 days. Additional exogenous recombinant S100A4 (see Data Sheet 1 in the Supplementary Material) dose-dependently reduced 5-FU-induced apoptosis in the S100A4^+^ MCS2 cell line (Figure 3E; Figure S1B in Supplementary Material). As observed for primary MDSCs, the effect of S100A4 could be blocked by the 3B11 antibody, which alone did not affect MSC2 viability (Figure 3F). Transmission electron microscopy revealed severe karyopyknosis with condensed and fragmented nuclei, cell shrinkage, vacuole formation, and undulations of the plasma membrane in MSC2 cells treated with 5-FU. Features like this were rarely observed in non-treated cells or MSC2 cells exposed to 5-FU in the presence of exogenous S100A4 (Figure 3G).

We concluded that increased MDSC apoptosis resulted in the impaired accumulation of MDSCs in tumor-bearing S100A4^−/−^mice. The finding that exogenous S100A4 enhanced the protective effect of endogenous S100A4 strengthens the contention that S100A4 is an important survival factor for MDSCs.

### S100A4 Protecting MDSCs from the Induction of Intrinsic Apoptosis

**Figure 4:**
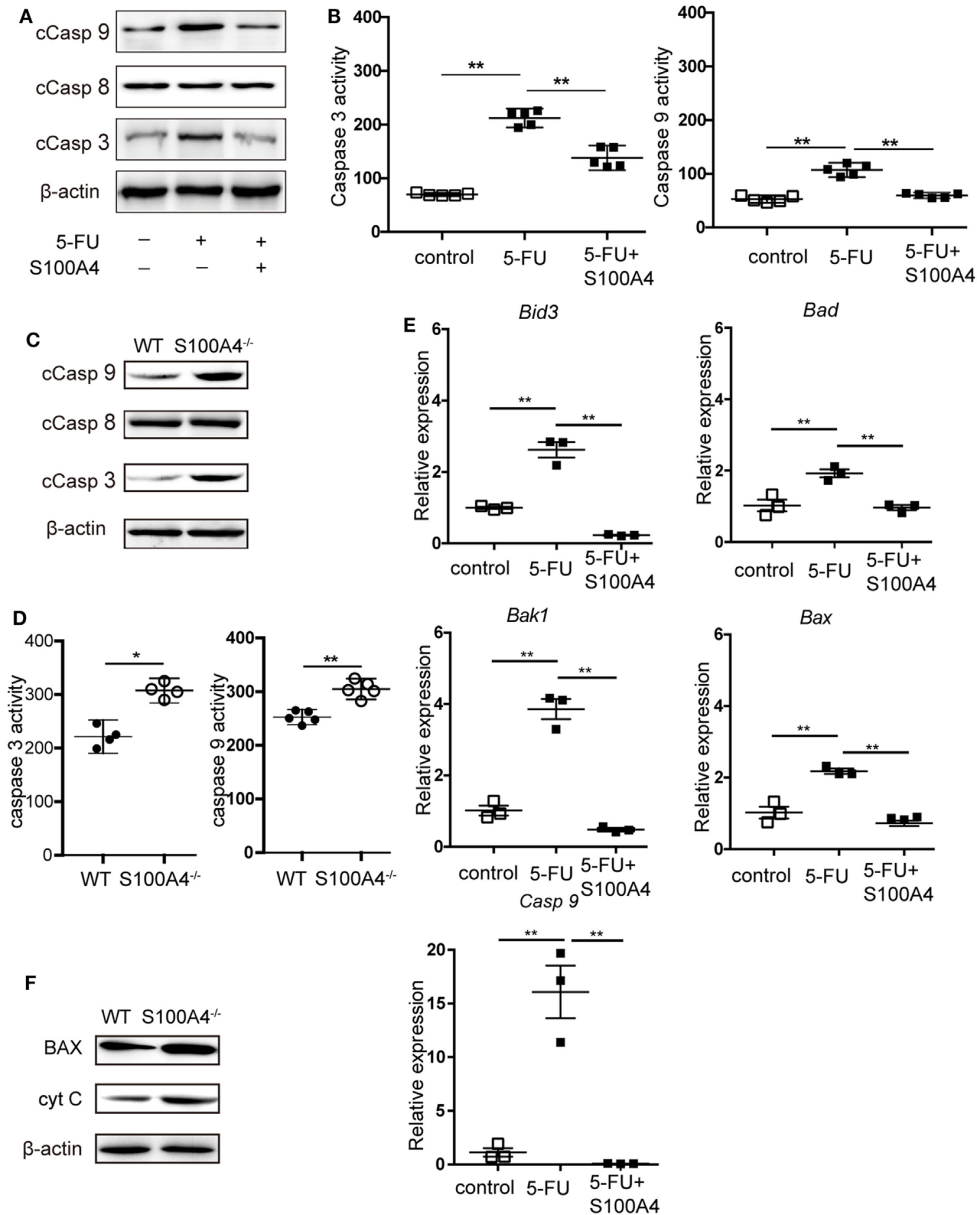
Effect of S100A4 on caspase activation and mitochondrial markers upon 5-FU-induced apoptosis in myeloid-derived suppressor cells (MDSCs). **(A)** MSC2 cells were treated with 5-fluorouracil (5-FU) or 5-FU and S100A4 (1 µg/mL) for 24 h; cleaved caspase (Casp)-3 (17 kDa), cleaved Casp-8 (18 kDa), and cleaved Casp-9 (37 kDa) proteins were evaluated by western-blot analysis. β-actin (42 kDa) served as a loading control. Representative images for three independent experiments are shown. **(B)** MSC2 cells were treated with 5-FU alone or in the presence of S100A4, as indicated. Cleaved caspase-3 and cleaved caspase-9 activities were determined by colorimetric activity assays. Representative results for three independent experiments are shown. Mean and 95% CI, *n* = 5 per group; **P < 0.01, Mann–Whitney. **(C)** Total cell lysates from purified MDSCs were subjected to western-blot analysis for cleaved caspase-3, cleaved caspase-8, or cleaved caspase-9. β-actin served as a loading control. Representative results for three independent experiments are shown. **(D)** Cleaved caspase-3 and cleaved caspase-9 activities from lysates of splenic CD11b^+^GR1^+^ cells from tumor-bearing wild type (WT) and S100A4^−/−^ mice were determined by colorimetric assays. Representative results of three independent experiments are shown. Mean and 95% CI, *n* = 4–5 per group; **P < 0.01, Mann–Whitney. **(E)** The mRNA expression levels of BH3 interacting-domain death agonist (*Bid3*), Bcl-2-associated death promoter (*Bad*), Bcl-2-associated X protein (*Bax*), Bcl-2 homologous antagonist/killer (*Bak1*), and caspase-9 (*Casp9*) in MSC2 cells were detected by quantitative RT-PCR. Expression was normalized to that of *Actb*. Representative results from three independent experiments are shown. Mean ± SEM, *n* = 3 per group; **P < 0.01, ANOVA. **(F)** Total cell lysates of splenic CD11b^+^GR1^+^ cells from tumor-bearing WT and S100A4^−/−^ mice were subjected to BAX (20 kDa) or cytochrome c (CytC; 12 kDa)-specific western-blot analysis. β-actin served as loading control. Representative results of three independent experiments are shown.

We further investigated how S100A4 is involved in protecting MDSCs from apoptosis. DNA fragmentation in 5-FU-treated MSC2 cells, as detected by TUNEL staining, was reduced from approximately 40 to 30% in the presence of recombinant S100A4 (Figure S3A in Supplementary Material). Upon assessing more upstream processes by confocal microscopy (Figure S3B in Supplementary Material) and flow cytometry (Figure S3C in Supplementary Material), we found that 5-FU-induced caspase-3 cleavage in MSC2 cells was partially abrogated through the addition endogenous S100A4. Again, in MSC2 cells, recombinant S100A4 prevented increases in cleaved caspase-3 and cleaved caspase-9, which was induced by 5-FU treatment. Cleaved caspase-8 levels remained unaffected in all groups (Figure 4A). Higher levels of cleaved caspase-3 and cleaved caspase-9 within MSC2 cells treated with 5-FU correlated with significantly higher activity of both enzymes, and this was reduced by the presence of recombinant S100A4 (Figure 4B).

When asking the same question using primary splenic MDSCs from MCA205 tumor-bearing WT or S100A4^−/−^ mice, only cleaved caspase-3 and cleaved caspase-9 were increased in S100A4^−/−^ MDSCs compared with levels in their WT counterparts (Figure 4C). Again, there was no difference in the cleavage of caspase-8. The higher cellular content of cleaved caspase-3 and cleaved caspase-9 in the S100A4^−/−^ MDSCs, compared with that in WT MDSCs, suggests increased enzymatic activities of both enzymes (Figure 4D). The association with caspase-9, but not caspase-8, cleavage suggested that S100A4 has a specific effect on the induction of intrinsic apoptosis (20). In further experiments, we tested the effect of recombinant S100A4 on the mRNA levels of mitochondria-related pro-apoptosis genes involved in intrinsic apoptosis in 5-FU-treated MSC2 cells. Significantly elevated expression of the genes encoding BH3 interacting-domain death agonist (*Bid3*), Bcl-2-associated X protein (*Bax*), Bcl-2 homologous antagonist/killer (*Bak1*), Bcl-2-associated death promoter (*Bad*), and caspase-9 was effectively reversed upon treatment with S100A4 (Figure 4E). Comparing BAX protein levels, as a representative protein, in primary MDSCs, enhanced expression was observed in MDSCs from S100A4^−/−^ mice compared with that in cells from WT mice, and the expression level of cytochrome c was also enhanced (Figure 4F). Taken together, these results demonstrated that S100A4 protects MDSCs from intrinsic apoptosis.

### Exogenous S100A4 Mediating Its Anti-Apoptotic Effect *via* TLR4

**Figure 5:**
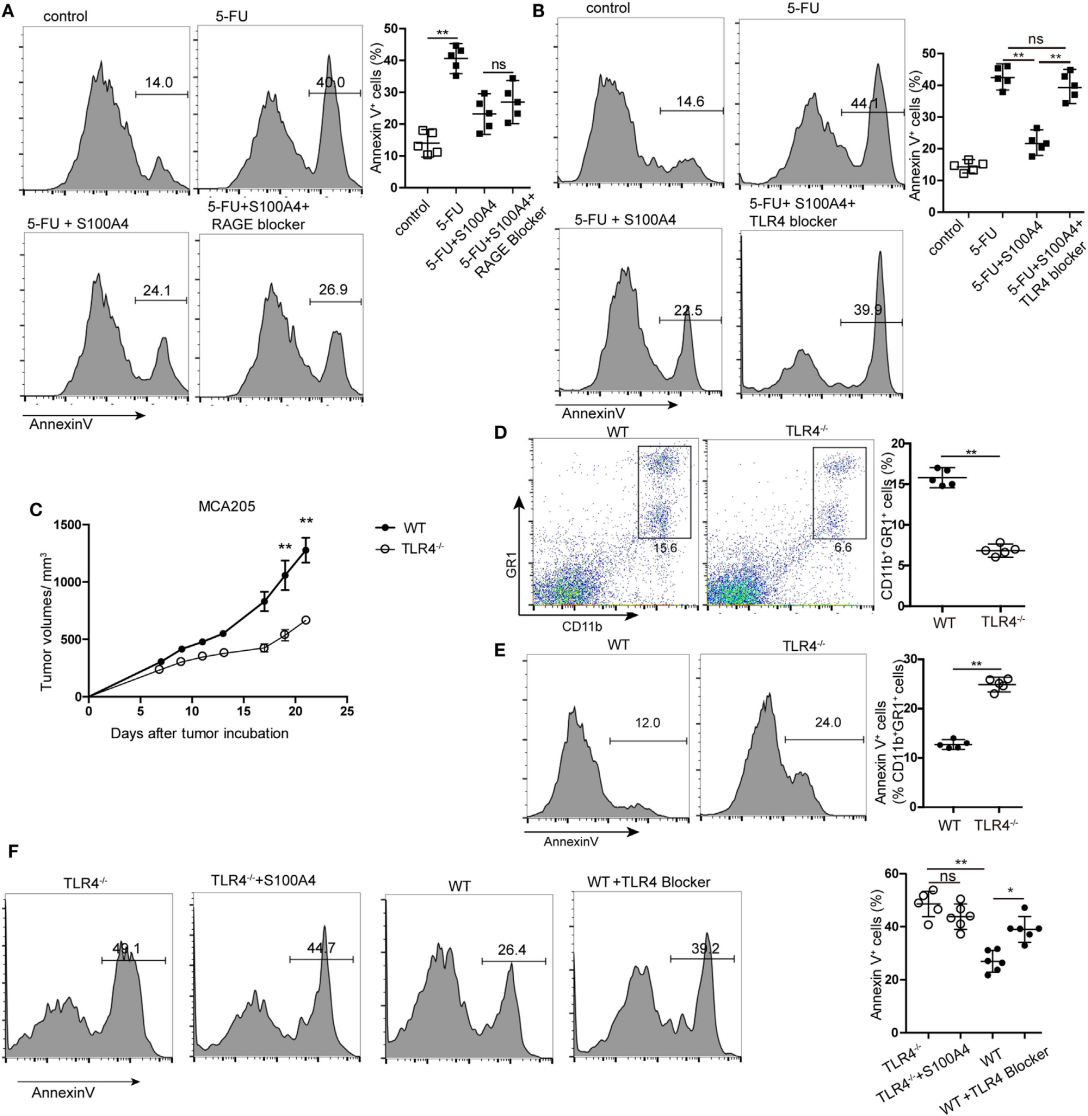
Receptor dependency of anti-apoptotic extracellular S100A4. **(A,B)** MSC2 cells were cultured with inhibitors of **(A)** receptor of advanced glycation end-products (RAGE) or **(B)** toll-like receptor-4 (TLR4) in the presence of 5-fluorouracil (5-FU) and S100A4, and the proportion of Annexin V^+^ cells was determined by flow cytometry. Mean and 95% CI, *n* = 5 per group; **P < 0.01, Mann–Whitney. **(C)** Wild type (WT) and TLR4^−/−^ mice were subcutaneously injected with 5 × 10^5^ MCA205 cells. Tumor volumes were monitored over time after tumor-cell inoculation. Representative results of three independent experiments are shown. Mean ± SEM, *n* = 5 per group; **P < 0.01, Mann–Whitney. **(D,E)** Spleen cells from tumor-bearing WT and TLR4^−/−^ mice stained for CD11b, GR1, and Annexin V were assessed by flow cytometry. Representative results of three independent experiments are shown. Mean and 95% CI, *n* = 5 per group; **P < 0.01, Mann–Whitney. **(F)** Myeloid-derived suppressor cells (MDSCs) were isolated from tumor-bearing TLR4^−/−^ or WT mice and cultured in the presence of S100A4 protein (1 µg/mL) alone or with a TLR4 inhibitor for 24 h. Cells were stained with Annexin V and assessed by flow cytometry. Combined raw data of two independent experiments are shown. Mean and 95% CI, *n* = 6 per group; *P < 0.05, **P < 0.01, and Mann–Whitney.

Extracellular S100A4 can signal *via* the receptor for advanced glycation end-product (RAGE) or *via* TLR4 (23, 24); thus, we evaluated the contribution of both receptors to the S100A4-mediated effect on intrinsic apoptosis in MDSCs. MSC2 cells preincubated with specific inhibitors of TLR4 or RAGE were cultured in the presence of 5-FU and soluble recombinant S100A4 was used as described above. Blocking TLR4 with VIPER completely abrogated the anti-apoptotic capacity of S100A4 within the cultures (Figure 5B), while blocking RAGE-mediated signaling by FPS-ZM1 did not alter the response of the cells to external S100A4 (Figure 5A). In addition, MSC2 cells constitutively express surface TLR4, which can be upregulated by exogenous S100A4 (Figure S4 in Supplementary Material). Therefore, we concluded that the effect of extracellular S100A4 on MDSCs was mainly mediated by TLR4.

Using TLR4 knockout (TLR4^−/−^) mice with the MCA205 tumor model confirmed the involvement of TLR4 in S100A4-dependent signaling *in vivo*. Comparable to results with S100A4^−/−^ mice (see Figures 1A,F), tumors in TLR4^−/−^ mice were significantly smaller than those in WT mice starting at day 17 after tumor-cell inoculation (Figure 5C). Percentages of CD11b^+^GR1^+^ cells in the spleens of TLR4^−/−^ mice bearing MCA205 tumors were also lower compared with those in WT controls (Figure 5D). In contrast to that observed with splenic MDSCs from mice lacking S100A4 (Figure 3B), TLR4^−/−^ MDSCs showed higher levels of apoptosis than WT MDSCs (Figure 5E). MDSCs isolated from the spleens of tumor-bearing WT mice (positive for S100A4) responded to the TLR4 inhibitor with significantly increased apoptosis compared with that in control MDSCs. Consequently, the addition of exogenous S100A4 to MDSC cultures isolated from TLR4^−/−^ mice did not rescue the cells from apoptosis (Figure 5F).

These *in vitro* and *in vivo* results demonstrated that extracellular S100A4 can act *via* TLR4 to protect MDSCs from apoptosis.

### S100A4–TLR4 Signaling in MDSCs Activating the Extracellular Signal-Regulated Kinase (ERK) 1/2 Pathway

**Figure 6:**
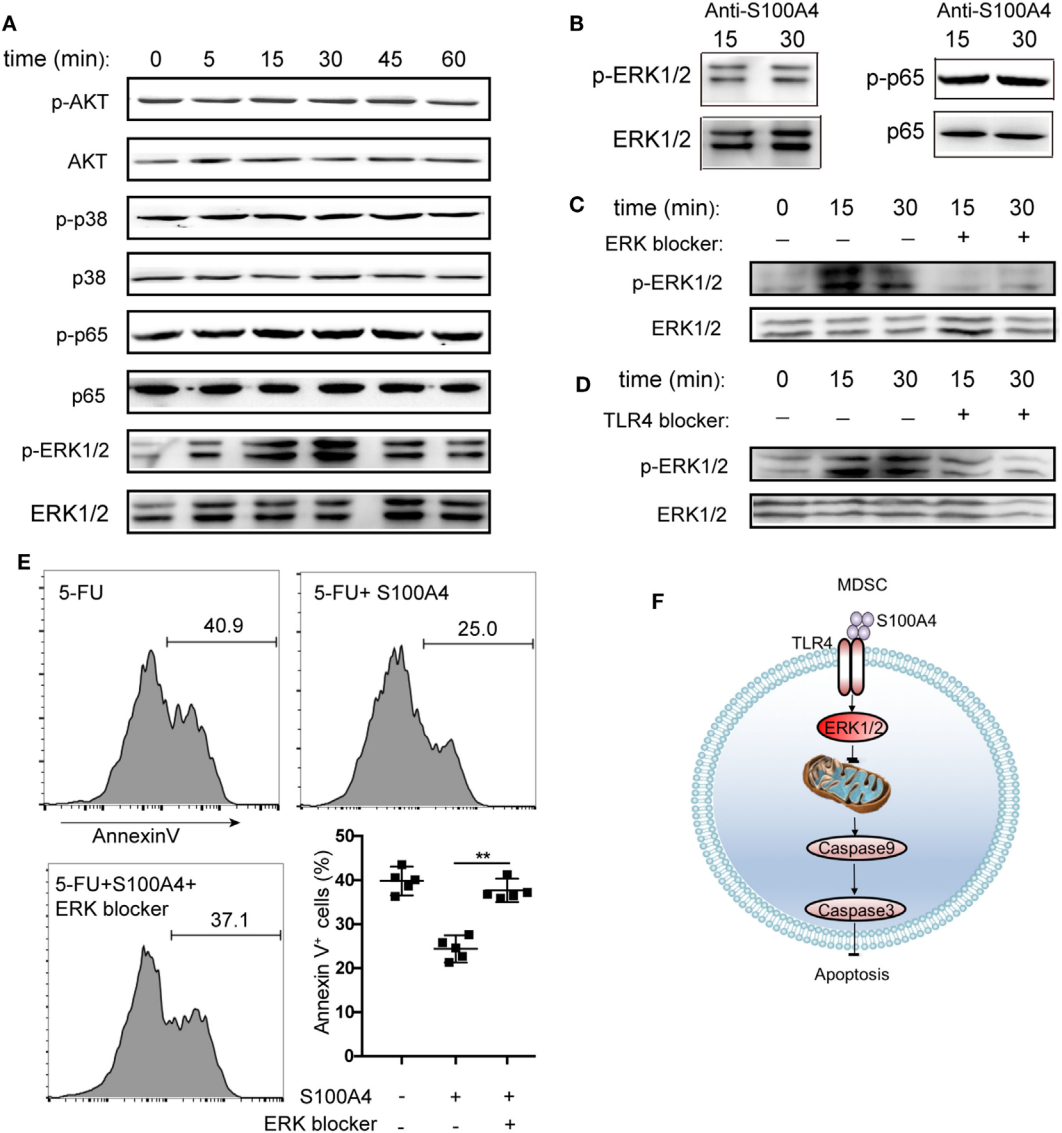
Activation of different kinase pathways by S100A4 in myeloid-derived suppressor cells (MDSCs). **(A)** MSC2 cells were cultured with S100A4 (1 µg/mL) for the indicated durations before phosphorylation of p65 of NFκB (65 kDa), AKT (56 kDa), p38 (38 kDa), or ERK1/2 (44 kDa, 42 kDa) was determined from cell lysates by western-blot analysis. Respective total protein served as loading control. **(B)** MCS2 cells were treated with S100A4 (1 µg/mL) in combination with the S100A4 neutralizing antibody, 3B11 (6 µg/mL), and subjected to western-blot analysis for (p-)p65 or (p-)ERK1/2. **(C,D)** MSC2 cells were treated with **(C)** extracellular signal-regulated kinase (ERK) (50-nM SCH772984) or **(D)** toll-like receptor-4 (TLR4) (10-µM VIPER) inhibitors for 30 min before the levels of phosphorylated ERK1/2 were determined from total cell lysates by western-blot analysis. **(A–D)** Representative results of three independent experiments are shown. **(E)** MSC2 cells were pretreated with an ERK1/2 inhibitor (50-nM SCH772984) for 30 min before S100A4 (1 µg/mL) was added. Cells were stained with Annexin V and assessed by flow cytometry. Representative results of three independent experiments are shown. Mean and 95% CI, *n* = 4 per group; **P < 0.01, Mann–Whitney. **(F)** Schematic summary for a role of S100A4-recruited TLR4–ERK1/2 signaling in protecting MDSCs from intrinsic apoptosis induction.

To determine the intracellular pathways involved in the inhibition of MDSC apoptosis by exogenous S100A4, we incubated primary splenic MDSCs from tumor-bearing mice with recombinant S100A4 and studied the early kinetics of activation of downstream pro-survival factors. S100A4 failed to activate the canonical myeloid differentiation primary response gene-88 (MyD88)-dependent TLR4 pathway, as shown by unaltered phosphorylation of the p65 subunit of nuclear factor (NF)-κB. Similarly, the kinases AKT and p38, also involved in cell survival, were not affected by exogenous S100A4. In contrast, the addition of S100A4 increased ERK1/2 phosphorylation, which peaked within approximately 30 min, and declined to nearly initial levels within 60 min (Figure 6A). Confirming the connection between ERK1/2 signaling and exogenous S100A4, preincubation of S100A4 with the neutralizing S100A4-specific antibody 3B11 inhibited the phosphorylation of ERK1/2 (Figure 6B), which was observed after 30 min without the antibody (see Figure 6A). The antibody had no effect on NFκB phosphorylation (Figure 6B). The presence of the ERK inhibitor SCH772984 reduced ERK1/2 phosphorylation at the time points of peak activation, after 15 and 30 min, to baseline levels, thus proving that S100A4 specifically activates ERK1/2 (Figure 6C).

To verify the importance of ERK1/2 after the interaction between exogenous S100A4 and TLR4, the effect of soluble, recombinant S100A4 on MSC2 cells on phosphorylated ERK1/2 was studied after exposing cells to the TLR4 inhibitor VIPER. Blocking TLR4 reduced the phosphorylation of ERK1/2 (Figure 6D), but this effect was not as apparent as that with direct inhibition of ERK1/2 (see Figure 6C). Consistently, S100A4 could not rescue MSC2 cells pretreated with an ERK1/2 inhibitor from 5-FU-induced apoptosis (Figure 6E).

We concluded that the activation of TLR4 by S100A4 and subsequent ERK1/2 signaling is crucial for inhibiting intrinsic apoptosis in MDSCs.

## Discussion

Addressing mechanisms that drive the accumulation of MDSCs during chronic immune activation, our study demonstrated that extracellular S100A4 protects MDSCs from mitochondria-dependent intrinsic apoptosis *via* TLR4-mediated ERK1/2 signaling. The key feature of our model was the diminished activation of caspase-9 in the presence of exogenous S100A4, which could be reverted by blocking the interaction between S100A4 and its receptor TLR4. Our results thus provide evidence for a new immunomodulatory function for S100A4.

Myeloid-derived suppressor cells evade apoptosis *via* several mechanisms during chronic inflammation. The abundance of MDSCs during chronic inflammation implies that these cells should have more than one mechanism to effectively counteract the various stimuli involved in apoptosis induction. The mitochondria-dependent intrinsic apoptosis pathway is activated by intracellular stress such as DNA damage, nutrient deprivation, ROS, and ER stress. MDSCs produce significant amounts of ROS and other small molecules that provide very effective signals to target immune cells and to control acute and chronic inflammation (34, 35). Intrinsic apoptosis induction is the common theme for the M-MDSCs or PMN-MDSCs subtypes that contribute to the overall immunosuppressive effect of a given MDSC population (36). To date, it was not known whether and how individual MDSCs can escape intrinsic apoptosis. Here, we found that S100A4 could be a main factor that protects MDSCs from intrinsic stress, and results suggested that this occurs *via* TLR4–ERK1/2 signaling. We previously showed that TNF contributes to the accumulation of MDSCs *via* TNF receptor-2-mediated inhibition of caspase-8 activity (7). Other mechanisms protecting MDSCs from extrinsic apoptosis include signaling *via* the interleukin-4 receptor (37) or decreased surface expression of FAS with diminished expression of interferon-regulatory factor-8 and proapoptotic BAX, as well as increased levels of the anti-apoptotic protein BCL-XL (15). It is not presently clear whether TNF, S100A4, or other factors mediate this function separately or synergistically during MDSC accumulation. Further understanding of how peripheral MDSCs evade apoptosis requires further study.

We decided to study the link between S100A4 and MDSCs based on the fact that high levels of exogenous S100A4 are a hallmark of tumor tissues (26, 27). S100A4 is a very versatile protein that is known to participate in cell survival and migration as well as angiogenesis (25). We previously found that during liver inflammation, macrophage-derived S100A4 activates collagen synthesis in hepatic stellate cells, promoting liver fibrosis and cancer development (38). Our recent work found that in the liver, extracellular S100A4 regulates the survival of CD8^+^ T cells by directly activating the AKT pathway. Nevertheless, how S100A4 affects inflammation in spleen, which is the main immune organ, is not known. Our present work mainly studied the pro-survival function of S100A4 in the spleen.

To specifically study intrinsic apoptosis, we used 5-FU, a prototypic inducer of this process. 5-FU induces apoptosis by inhibiting thymidylate synthase and the incorporation of its metabolites into DNA to disrupt DNA synthesis and repair (39). Chemotherapy with 5-FU effectively depletes peripheral MDSCs. After the depletion of MDSC numbers, these cells can recover and later contribute to tumor growth, as shown in a mouse EL4 lymphoma model (40). The marked effect of exogenous S100A4 on protecting MDSCs from 5-FU-induced DNA damage (as shown in the present study) suggested that S100A4 is a crucial survival factor for these immunomodulatory cells and indicated that an S100A4 neutralizing antibody might augment 5-FU-based anticancer chemotherapy.

Extracellular S100A4 signals *via* TLR4, defining a function for this receptor in sterile inflammatory conditions. TLR4 was long thought to be exclusively involved in innate immune responses, protecting the host from invading microbial pathogens, and as a prerequisite for the subsequent induction of adaptive immune responses during tissue damage or inflammation (41, 42). Meanwhile, TLR signals were also found to regulate B-cell activation and survival (43). It has been reported that TLR4 signaling enhances MDSC-mediated immune suppression. Through TLR4 signaling, MDSC produce IL-10 and downregulate macrophage production of IL-12 (9). We provide new evidence suggesting that S100A4 is a ligand of TLR4, and we suggest a mechanism that S100A4 protects MDSC from intrinsic apoptosis *via* TLR4 signaling. This demonstrates that TLR4 is not only involved in promoting inflammation, but also participates in the cell-mediated regulation of ongoing immune responses. Whether the binding of S100A4 to TLR4 alone is sufficient for this signaling or whether other membrane molecules are recruited upon binding needs to be elucidated.

Our key findings are as follows: (i) deletion or inhibition of S100A4 has a proapoptotic effect on MDSCs, (ii) excess exogenous S100A4 inhibits 5-FU-induced MDSC apoptosis, and (iii) the absence of the S100A4 surface receptor, TLR4, increases MDSC apoptosis. These results suggest that targeting extracellular S100A4 could represent a therapeutic option to control chronic inflammation and tumorigenesis. Regarding the clinical implications of exogenous S100A4 as an important survival factor for MDSCs, caution is required. S100A4 is part of a highly conserved family of small proteins, suggesting critical functions in mechanisms not related to MDSC persistence.

In addition to our study of the role of S100A4 in regulating MDSC apoptosis, multiple reports have focused on its role in cancer progression, specifically its ability to enhance metastasis. Furthermore, recently, previous studies by ourselves and other authors have linked S100A4 to several diseases besides cancer, including kidney fibrosis (44, 45), pulmonary disease (46, 47), cardiac hypertrophy and fibrosis (48, 49), arthritis (50), and neuronal injuries (51). S100A4 was also shown to be a prognostic marker for kidney disease. Our work supports the use of S100A4 not only as a disease marker but also as a drug target.

Collectively, our data suggest that the inhibition of intrinsic apoptosis in MDSCs by the S100A4–TLR4–ERK1/2 signaling axis is an important and novel regulatory mechanism that allows MDSCs to persist (Figure 6F). These findings also highlight a role for TLR4 during aseptic inflammatory conditions and especially link extracellular S100A4 to mechanisms used by MDSCs to escape the danger of low molecular weight effector molecules that are part of a pro-inflammatory environment and are also produced by the MDSCs themselves. Understanding how diverse mechanisms that regulate proliferation and the prevention of apoptosis synergize to maintain MDSC accumulation during chronic inflammation and tumorigenesis will help to uncover novel strategies for the therapeutic targeting of S100A4.

## Author Contributions

QL, CD, LC, and ZQ conceived the study and designed the experiments. QL, CD, PW, UE, and ZQ analyzed and interpreted the data. QL, CD, RX, PW, UE, YH, and ZQ wrote the manuscript.

## Conflict of Interest Statement

The authors declare that the research was conducted in the absence of any commercial or financial relationships that could be construed as a potential conflict of interest.

## Supplementary Material

Data Sheet 1 Supplementary method.

Figure S1 Expression of S100A4 in murine tumors and myeloid-derived suppressor cell (MDSC) cell lines. Proteins from total cell lysates of 1 × 10^6^ **(A)** MCA205, Lewis lung cancer (LLC), or B16F10 tumor cells or **(B)** MSC2 MDSCs from standard tissue culture were separated by SDS-PAGE. Levels of S100A4 (12 kDa), relative to those of β-actin (42 kDa) as a loading control, were assessed by western blot. Images are representative of three independent experiments.

Figure S2 Composition of the immune-cell compartment within the spleens of MCA205 tumor-bearing mice. Mice were subcutaneously injected with 5 × 10^5^ MCA205 tumor cells. On day 17 after tumor induction, splenic cells were stained for CD11c, B220, NK1.1, CD8, CD4, and Foxp3 and analyzed by flow cytometry. Shown are percentages of indicated cells in wild type (WT) and S100A4^−/−^ mice. Representative results of three independent experiments are shown. Mean and 95% CI, *n* = 5 per group.

Figure S3 Detection of apoptosis in MSC2 cells treated with exogenous S100A4. MSC2 cells were treated with 5-fluorouracil (5-FU) alone, 5-FU in combination with S100A4 (1 ng/mL), or were left untreated for 24 h. **(A)** DNA fragmentation of MSC2 cells was detected by flow cytometry after TUNEL. Representative results of three independent experiments are shown. Mann–Whitney, mean, and 95% CI, *n* = 5 per group. ***P* < 0.01. **(B,C)** Cells stained with DAPI and caspase-3 were analyzed by **(B)** confocal microscopy or **(C)** flow cytometry. **(B)** Cultured cells were subjected to immunohistochemistry. Representative images from two independent experiments are shown. Scale bars: 25 µm. **(C)** Representative results for three independent experiments are shown. ANOVA, mean, and 95% CI, *n* = 5 per group. ***P* < 0.01.

Figure S4 Effect of exogenous S100A4 on toll-like receptor-4 (TLR4) expression in MSC2 cells. MSC2 cells treated for 60 min with exogenous S100A4 (1 µg/mL) were stained using TLR4-specific antibody (MTS510, eBioscience) and monitored by flow cytometry. Representative results for three independent experiments are shown. Mann–Whitney, mean, and 95% CI, *n* = 4 per group.
