# Supplementary material for: S100A4 Protects Myeloid-Derived Suppressor Cells from Intrinsic Apoptosis via TLR4–ERK1/2 Signaling
Source: Front Immunol. 2018 Mar 5;9:388. doi: 10.3389/fimmu.2018.00388 (PMC5845385; doi:10.3389/fimmu.2018.00388)
Supplement: Data Sheet 1 — Supplementary method. [file Data_Sheet_1.docx]

Recombinant S100A4. The S100A4 cDNA cloned into the bacterial expression vector pET28a (Novagen) was used to transform the Escherichia coli Rosetta producer strain (TransGene Biotech). His-tagged S100A4 protein was purified from lysed bacteria using Ni-Sepharose 6 FF column affinity chromatography (GE Healthcare) followed by a hollow fiber membrane ultrafiltration step to reduce bacterial endotoxins (GE Healthcare). Endotoxin concentrations of the final product were <1 EU/mL as confirmed by a Tachypleus tridentatus-based Gel Clot TAL Endpoint Assay Kit (Xiamen Bioendo Technology). The purity of S100A4 in the preparations was confirmed by SDS-PAGE and western blot.

S100A4-specific antibody. The neutralizing S100A4-specific antibody (3B11) was generated by immunizing BALB/c mice with the recombinant S100A4 protein. Using splenic cells and the mouse myeloma Sp2/0 cell line as a fusion partner, hybridomas were generated and selected using standard techniques [McEver RP, Baenziger NL, Majerus PW. Isolation and quantitation of the platelet membrane glycoprotein deficient in thrombasthenia using a monoclonal hybridoma antibody. J Clin Invest (1980) 66:1311–8. [doi:10.1172/JCI109983]. Antibodies were purified from the hybridoma culture supernatant by Protein-A affinity chromatography according to the manufacturer’s protocol (GE Healthcare). Purity and antibody concentrations in the preparation were assessed by SDS-PAGE.
