# Supplementary figures and images for: S100A4 Protects Myeloid-Derived Suppressor Cells from Intrinsic Apoptosis via TLR4–ERK1/2 Signaling
Source: Front Immunol. 2018 Mar 5;9:388. doi: 10.3389/fimmu.2018.00388 (PMC5845385; doi:10.3389/fimmu.2018.00388)

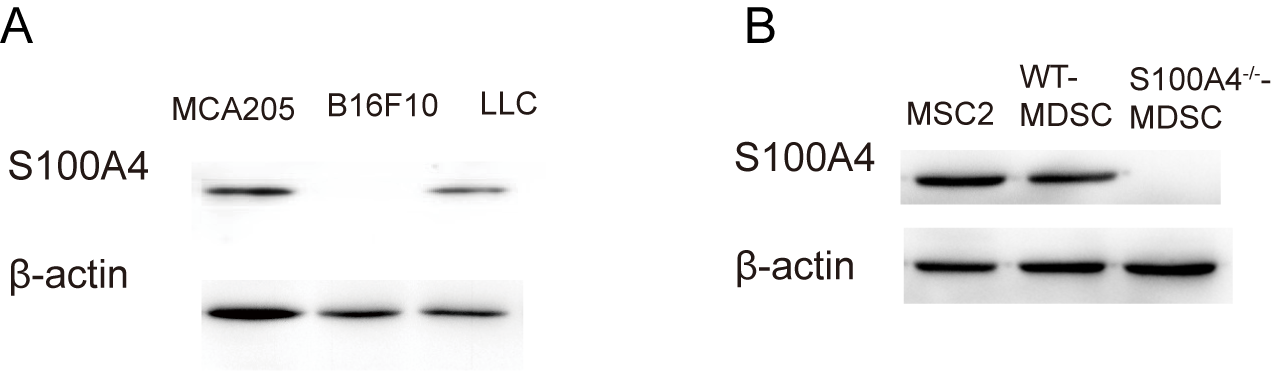

Supplement: Figure S1 — Expression of S100A4 in murine tumors and myeloid-derived suppressor cell (MDSC) cell lines. Proteins from total cell lysates of 1 × 10^6^ (A) MCA205, Lewis lung cancer (LLC), or B16F10 tumor cells or (B) MSC2 MDSCs from standard tissue culture were separated by SDS-PAGE. Levels of S100A4 (12 kDa), relative to those of β-actin (42 kDa) as a loading control, were assessed by western blot. Images are representative of three independent experiments. [file image_1.tif]

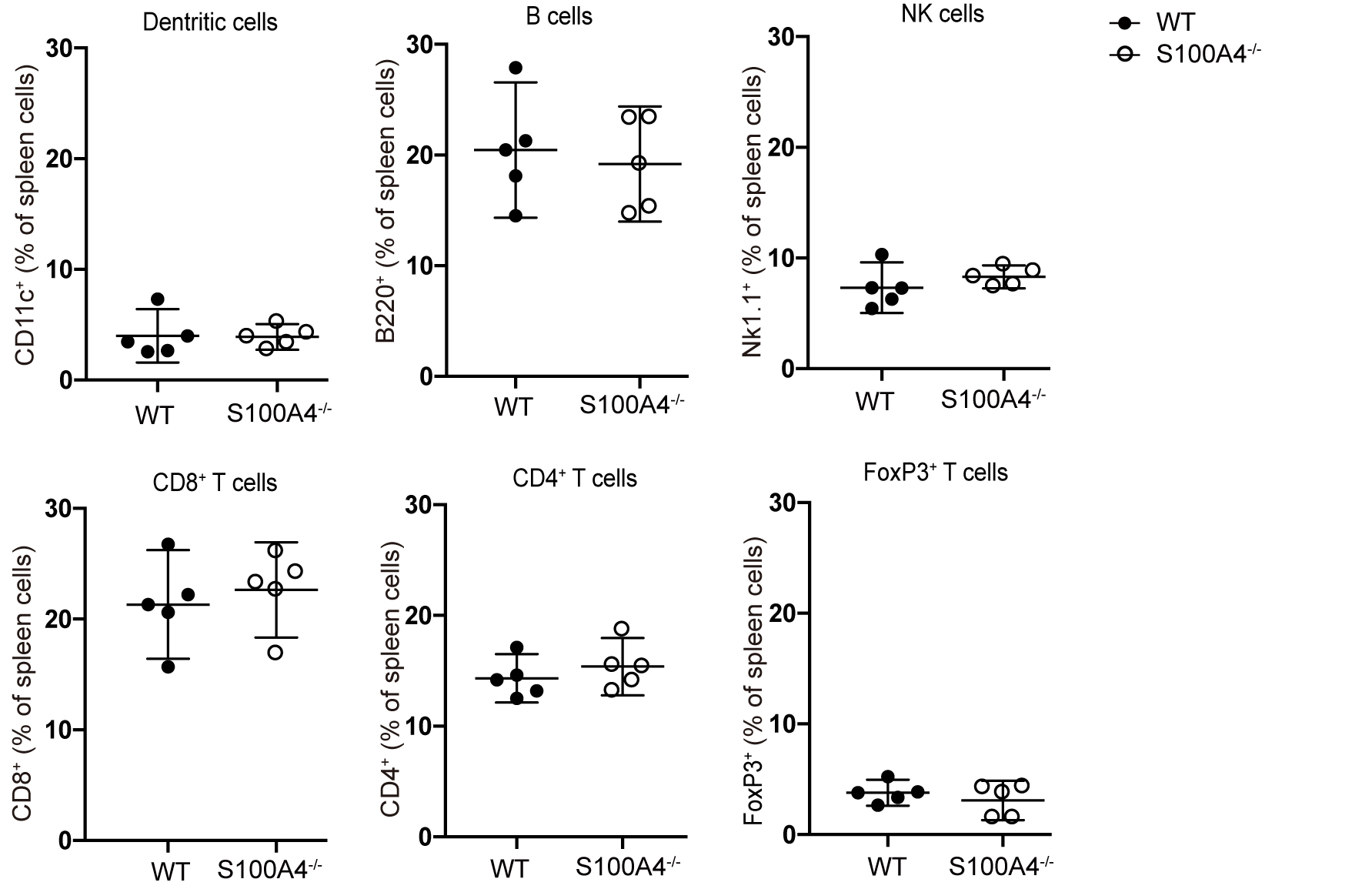

Supplement: Figure S2 — Composition of the immune-cell compartment within the spleens of MCA205 tumor-bearing mice. Mice were subcutaneously injected with 5 × 10^5^ MCA205 tumor cells. On day 17 after tumor induction, splenic cells were stained for CD11c, B220, NK1.1, CD8, CD4, and Foxp3 and analyzed by flow cytometry. Shown are percentages of indicated cells in wild type (WT) and S100A4−/− mice. Representative results of three independent experiments are shown. Mean and 95% CI, n = 5 per group. [file image_2.jpg]

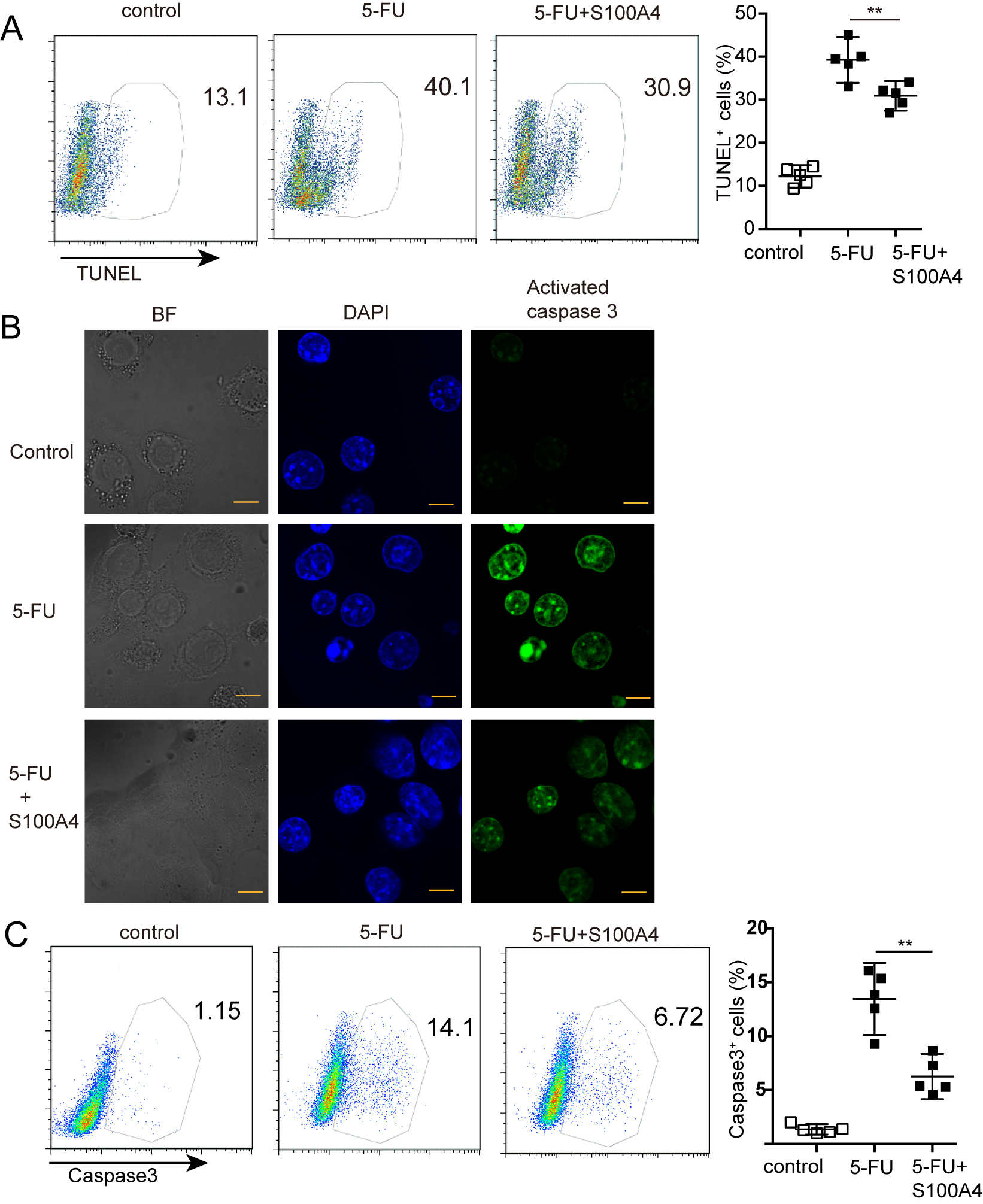

Supplement: Figure S3 — Detection of apoptosis in MSC2 cells treated with exogenous S100A4. MSC2 cells were treated with 5-fluorouracil (5-FU) alone, 5-FU in combination with S100A4 (1 ng/mL), or were left untreated for 24 h. (A) DNA fragmentation of MSC2 cells was detected by flow cytometry after TUNEL. Representative results of three independent experiments are shown. Mann–Whitney, mean, and 95% CI, n = 5 per group. **P < 0.01. (B,C) Cells stained with DAPI and caspase-3 were analyzed by (B) confocal microscopy or (C) flow cytometry. (B) Cultured cells were subjected to immunohistochemistry. Representative images from two independent experiments are shown. Scale bars: 25 µm. (C) Representative results for three independent experiments are shown. ANOVA, mean, and 95% CI, n = 5 per group. **P < 0.01. [file image_3.jpg]

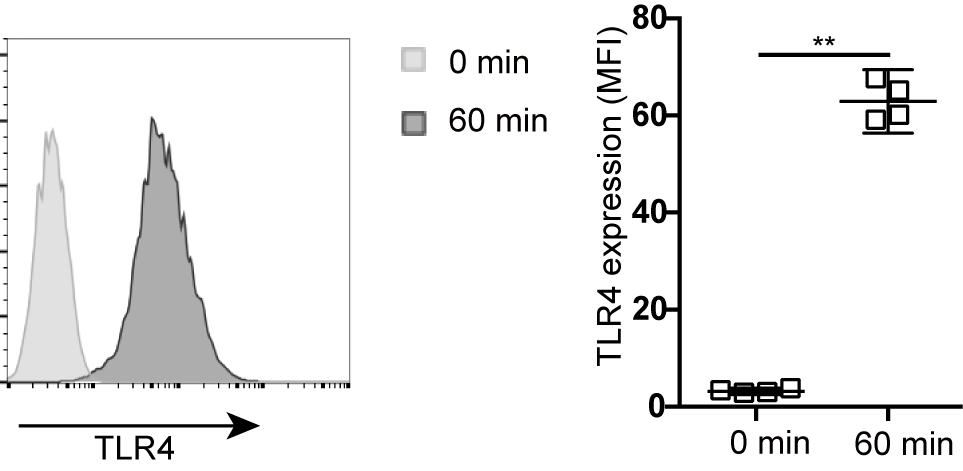

Supplement: Figure S4 — Effect of exogenous S100A4 on toll-like receptor-4 (TLR4) expression in MSC2 cells. MSC2 cells treated for 60 min with exogenous S100A4 (1 µg/mL) were stained using TLR4-specific antibody (MTS510, eBioscience) and monitored by flow cytometry. Representative results for three independent experiments are shown. Mann–Whitney, mean, and 95% CI, n = 4 per group. [file image_4.tif]
